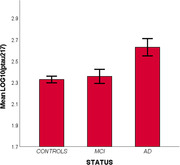# p‐tau217 in a large Peruvian cohort phenotyped for Alzheimer’s Disease

**DOI:** 10.1002/alz.093382

**Published:** 2025-01-09

**Authors:** Neetesh Pandey, Lawrence S. Honig, Dolly Reyes‐Dumeyer, Basilio Cieza, Rosa Montesinos, Jennifer Cespedes, Marcio F. Soto‐Añari, Nilton Custodio, Giuseppe Tosto

**Affiliations:** ^1^ Columbia University, New York, NY USA; ^2^ Taub Institute for Research on Alzheimer’s Disease and the Aging Brain, Vagelos College of Physicians and Surgeons, Columbia University, New York, NY USA; ^3^ Columbia University Irving Medical Center, New York, NY USA; ^4^ Unit Cognitive Impairment and Dementia Prevention, Peruvian Institute of Neurosciences, Lima, Peru, Lima, Lima Peru; ^5^ Universidad Católica San Pablo, Arequipa Peru; ^6^ Cognitive Impairment Diagnosis and Dementia Prevention Unit, Peruvian Institute of Neurosciences, Lima, Lima Peru

## Abstract

**Background:**

Blood‐based Alzheimer's disease (AD) biomarkers have been increasingly employed for diagnostic and prognostic purposes, thanks to high diagnostic accuracy in distinguishing AD from healthy controls or other dementia types. p‐tau217 exhibits stronger associations with AD hallmarks in CSF and brain, compared to other p‐tau isoforms. Furthermore, the majority of these studies have been conducted in non‐Hispanic Whites, limiting our understanding of the performances and utility of these biomarkers across ethnicities.

**Method:**

We employed a cohort of Peruvians from the GAPP study, a recently‐established cohort of Peruvian mestizos from Lima and indigenous groups from Southern Peru (Aymaras and Quechuas). We generated p‐tau ALZPATH217 in 561 samples, and tested the association between p‐tau217 and clinical diagnosis (healthy controls n=359 vs. AD n=92) using regression models adjusting for sex, age, education and study site. We also explored the biomarkers level in MCI (n=100). The receiver operating characteristics area under the curve (ROC‐AUC) was used to evaluate the biomarker’s classification performances.

**Result:**

p‐tau217 was significantly associated with AD (higher quartile vs. lower quartile: OR=6.49 95%CI 2.91‐14.90) while it did not differ between controls and MCI (p=0.33; Figure 1). p‐tau217 levels were also increasingly higher in participants carrying one or two APOE‐e4 alleles (ANOVA p‐value<0.001). When classification accuracy was considered, the resulting ROC area for p‐tau217 was 74%.

**Conclusion:**

To our knowledge, this is the largest study conducted in a Hispanic cohort phenotyped for AD with available p‐tau217. We confirmed the robust performances of the blood biomarker in a non‐White population; studies on Hispanic individuals with clinical AD diagnosis reported AUC as low as 52% vs. our robust finding of 74%. Previous investigations have mostly focused on highly selected cohorts with established AD‐endophenotypes (CSF, autopsies, PET etc.), while data on population cohorts with clinical assessment are currently lacking, especially in minoritized groups.